# Failure of a non-authorized copy product to maintain response achieved with imatinib in a patient with chronic phase chronic myeloid leukemia: a case report

**DOI:** 10.1186/1752-1947-3-7112

**Published:** 2009-04-29

**Authors:** Hadi Alphonse Goubran

**Affiliations:** 1 Professor of Medicine and Clinical Haematology, Faculty of Medicine, Cairo University, Maadi, 1431, Cairo, Egypt

## Abstract

**Introduction:**

Due to high rates of response and durable remissions, imatinib (Glivec^®^, or Gleevec^®^ in the USA; Novartis Pharma AG) is the standard of care in patients with chronic myeloid leukemia. Recently, a non-authorized product which claims comparability to imatinib has become available.

**Case presentation:**

This report describes the loss of response in a 36-year-old male patient with chronic-phase chronic myeloid leukemia who had previously been in full hematologic and cytogenetic remission and partial molecular remission for three years, under treatment with brand-name imatinib of 400 mg per day. Before the initiation of treatment with a copy product, imatib (CIPLA-India), the patient had negative BCR-ABL status. Within three months of initiation of treatment with the copy product, the patient's BCR-ABL status became positive, with substantial decreases noted in white blood cell counts, red blood cell counts and platelet counts. Conversion of the BCR-ABL status to negative and improvements in hematologic parameters were achieved when the brand medication, imatinib, was resumed at a dose of 600 mg per day.

**Conclusion:**

In our patient, the substitution of a copy product for imatinib resulted in the rapid loss of a previously stable response, with the risk of progression to life-threatening accelerated phase or blast crisis phase of the disease. Without supportive clinical evidence of efficacy and safety of imatib (or any other copy product) caution should be used when substituting imatinib in the treatment of any patient with chronic myeloid leukemia.

## Introduction

Imatinib, a potent and selective inhibitor of the protein tyrosine kinase BCR-ABL, has shown significant clinical activity in chronic myeloid leukemia (CML), producing durable responses and prolonged survival [1,2]. As such, imatinib (Glivec®, or Gleevec® in the US; Novartis Pharma AG) is considered the first-line standard of care for the treatment of patients with CML. Response to imatinib is assessed by hematologic white blood cell count (WBC) and platelet count; presence of extramedullary disease or leukemic cells in the peripheral blood, cytogenetic, percentage of Philadelphia chromosome-positive cells in the bone marrow, and molecular ratio of BCR-ABL to BCR transcripts in the peripheral blood as measured by real-time polymerase chain reaction [RT-PCR] parameters [1]. Long-term outcomes include freedom from progression to advanced phases of the disease and survival.

In health-care systems where patients have to pay for their own medicine, the cost of the medication can be an obstacle to adequate compliance. In life-threatening diseases, such as CML, a lapse in therapy may result in suboptimal responses, progression to accelerated or blast crisis phases of disease and mortality. Therefore, maintenance of response is of utmost importance.

A copy preparation, consisting of the alpha crystal form of imatinib, has become commercially available under the name 'imatib' (CIPLA-India) at a markedly reduced price. The lower price has prompted some health-care authorities and insurance payers to substitute the copy for imatinib. The manufacturer of imatib lists the product as being 'comparable' to imatinib [3], but the US regulatory authority the Food and Drug Administration (FDA) Orange Book does not have a listing of any products with equivalence to imatinib.

## Case presentation

A self-sponsored (that is, uninsured) 36-year-old man diagnosed with chronic-phase (CP) CML (Sokal score 1.22 [low risk <0.8, high risk >1.2] and Hasford score 1158.6 [low risk <780, moderate 780 to 1480, high risk >1480]) had received imatinib 400 mg per day since the beginning of January 2004. After approximately 10 months of therapy, full hematologic and cytogenetic remission, then major molecular remission had been achieved with a %BCR-ABL/ABL International Scale (IS) score of about 0.01 compared to an initial value of 9. This status had been maintained for approximately three years. In January 2007, his BCR-ABL scored negative by nested RT-PCR.

Subsequently, the patient sought medical insurance and was switched by the insurer to the copy product imatib, at the same dose of 400 mg per day. In March 2007, after approximately three months of therapy with the 'copy' imatib, and despite a claimed compliance to the prescribed regimen, substantial hematologic changes were noted in the form of anemia (hemoglobin 9 g/dL), neutropenia (WBC 2300/mm^3^ with an absolute neutrophil count (ANC) of below 1000), thrombocytopenia (platelets 101,000/mm^3^) and loss of cytogenetic and molecular responses. Cytogenetic and molecular assessments demonstrated a 'positive' Philadelphia chromosome (22% Ph+ cells) and a %BCR-ABL/ABL ratio of 3, consistent with a disease relapse and a probable hematological toxicity to the agent. The spleen was still impalpable and the Sokal score was 1.16. Tests for tyrosine kinase mutations were not performed.

In May 2007, the patient resumed imatinib at a daily dose of 600 mg per day. In July 2007, after approximately two months of therapy with imatinib, laboratory values revealed a return to a full hematologic and cytogenetic response and in November 2007, after six months of therapy, the patient achieved a %BCR-ABL/ABL score of 0.01. Changes in hematologic laboratory parameters at baseline, following the change to the copy product with a loss of response and subsequent return to treatment with imatinib and reinstatement of response, are summarized in Table 1.

**Table 1:** Hematologic parameters at baseline, following change to imatib and return to imatinib therapy

Laboratory value	January 2007Imatinib 400 mg/day	March 2007Imatib 400 mg/day	July 2007Imatinib 600 mg/day
Red blood cell count (n/mm^3^)	4,850,000	3,200,000	4,200,000
Hemoglobin (g/dL)	13.4	9	12.7
Total leukocyte count (n/mm^3^)	4200	2300	3800
Neutrophils	68%	34%	66%
Eosinophils	1%	7%	2%
Basophils	1%	8%	2%
Lymphocytes	24%	41%	25%
Monocytes	6%	10%	5%
Platelet count (n/mm^3^)	234,000	101,000	156,000

## Discussion

In the International Randomized Trial of Interferon versus STI571 (IRIS), responses to first-line imatinib have been remarkably durable [2]. Estimated annual rates of treatment failure after the start of imatinib therapy were 3.3% in the first year, 7.5% in the second year and 4.8% in the third year. It is possible that the rapid loss of response that followed the initiation of the copy product could have been coincidentally due to treatment failure associated with imatinib. However, the chronologic sequence of events points to a direct relationship between the initiation of the copy product and the loss of response followed by improvement in hematologic status and return to a negative BCR-ABL status that occurred after imatinib was restarted. Improvement in cytogenetic response following dose escalation of imatinib has been documented in patients with poor initial response or treatment failure [4]-[7]. In our patient, due to the rapid loss of response, the copy product was discontinued and imatinib was reinstated at an escalated dose. Without clinical trial data or documented clinical experience, it was not possible to know whether the rapid loss of response may have been attenuated or reversed by increasing the dose of the copy product.

Copy products, such as that described in our patient, should not be confused with generic equivalents to branded pharmaceutical products. In the US, a generic drug product is one that is comparable to an innovator drug product in dosage form, strength, route of administration, quality, performance characteristics (bioequivalence) and intended use [8]. Copy products often differ slightly in chemical structure and do not undergo clinical-trial evaluation or regulatory review before their release to market. Many of these products are offered for sale without prescription through internet-based distributors.

## Conclusion

While the copy product used by our patient is purported to be comparable to imatinib, there is no published clinical evidence that we are aware of to support this claim. In our patient, substitution of the copy product for imatinib resulted in rapid loss of a previously stable response. Loss of response in CML can result in progression to life-threatening accelerated phase or blast crisis phase of the disease.

The advent of therapies targeting tyrosine kinase resulted in a breakthrough in the management of CML. For cost reasons, some third-party insurers opt to use copy forms of the drug that might not be as efficacious as the original molecule. Every effort should be made to maintain patients in remission on their proper medicine, and careful follow-up is needed if patients who have achieved remissions are shifted to copy medicines, to ensure that the response is not compromised. Without supportive clinical evidence of efficacy and safety of this (or any other) copy product caution should be used in substitution of imatinib in any patient with CML.

## Abbreviations

BCR-ABL: breakpoint cluster region Abelson murine leukemia; CML: chronic myeloid leukemia; FDA: US Food and Drug Administration; CP: chronic phase; WBC: white blood cell count; ANC: absolute neutrophil count; RT-PCR: real-time polymerase chain reaction; IRIS: International Randomized Trial of Interferon versus STI571.

## Consent

Written informed consent was obtained from the patient for publication of this case report. A copy of the written consent is available for review by the Editor-in-chief of this journal.

## Competing interests

The author declares that he has no competing interests.

## Authors' contributions

HAG analyzed and interpreted the patient data regarding response and wrote and approved the manuscript.
